# The majority of microorganisms in gas hydrate-bearing subseafloor sediments ferment macromolecules

**DOI:** 10.1186/s40168-023-01482-5

**Published:** 2023-03-02

**Authors:** Chuwen Zhang, Yun-Xin Fang, Xiuran Yin, Hongfei Lai, Zenggui Kuang, Tianxueyu Zhang, Xiang-Po Xu, Gunter Wegener, Jiang-Hai Wang, Xiyang Dong

**Affiliations:** 1 School of Marine Sciences, Sun Yat-Sen University, Zhuhai, China; 2 Key Laboratory of Marine Genetic Resources, Third Institute of Oceanography, Ministry of Natural Resources, Xiamen, China; 3 Guangzhou Marine Geological Survey, China Geological Survey, Ministry of Natural Resources, Guangzhou, China; 4 Faculty of Biology/Chemistry, University of Bremen, Bremen, Germany; 5 MARUM, Center for Marine Environmental Sciences, University of Bremen, Bremen, Germany; 6 Max Planck Institute for Marine Microbiology, Bremen, Germany; 7 Southern Marine Science and Engineering Guangdong Laboratory (Zhuhai), Zhuhai, China

## Abstract

**Background:**

Gas hydrate-bearing subseafloor sediments harbor a large number of microorganisms. Within these sediments, organic matter and upward-migrating methane are important carbon and energy sources fueling a light-independent biosphere. However, the type of metabolism that dominates the deep subseafloor of the gas hydrate zone is poorly constrained. Here we studied the microbial communities in gas hydrate-rich sediments up to 49 m below the seafloor recovered by drilling in the South China Sea. We focused on distinct geochemical conditions and performed metagenomic and metatranscriptomic analyses to characterize microbial communities and their role in carbon mineralization.

**Results:**

Comparative microbial community analysis revealed that samples above and in sulfate-methane interface (SMI) zones were clearly distinguished from those below the SMI. Chloroflexota were most abundant above the SMI, whereas Caldatribacteriota dominated below the SMI. Verrucomicrobiota, Bathyarchaeia, and Hadarchaeota were similarly present in both types of sediment. The genomic inventory and transcriptional activity suggest an important role in the fermentation of macromolecules. In contrast, sulfate reducers and methanogens that catalyze the consumption or production of commonly observed chemical compounds in sediments are rare. Methanotrophs and alkanotrophs that anaerobically grow on alkanes were also identified to be at low abundances. The ANME-1 group actively thrived in or slightly below the current SMI. Members from Heimdallarchaeia were found to encode the potential for anaerobic oxidation of short-chain hydrocarbons.

**Conclusions:**

These findings indicate that the fermentation of macromolecules is the predominant energy source for microorganisms in deep subseafloor sediments that are experiencing upward methane fluxes.

Video Abstract

**Supplementary Information:**

The online version contains supplementary material available at 10.1186/s40168-023-01482-5.

## Background

Gas hydrates, ice-like crystalline solids composed of water and hydrocarbons, are widely discovered in the deep subseafloor of every continental margin [1, 2], typically hundreds of meters below the seafloor, e.g., the Hydrate Ridge [3] and Nankai Trough [4, 5]. Ecological studies based on single marker genes have revealed abundant and diversified members of archaea and bacteria in the deep subsurface sediments associated with gas hydrates over the global ocean [3–7]. All known forms of life require sources of carbon and energy to thrive. The deep subseafloor ecosystem is highly energy-limited, and microbial metabolic rates are among the lowest known on Earth [8, 9]. How microorganisms acquire carbon and energy sources in deep subseafloor sediments in the gas hydrate zone remains elusive.

Marine sediments contain Earth’s largest pool of organic carbon, derived from primary production in the overlying water column, land-derived inputs, cell debris after lysis and death, or exudates [10, 11]. Based on studies in various oceanic regions, e.g., the Guaymas Basin [12, 13], Eastern Gulf of Mexico [14], and Helgoland mud area [15, 16], the vast majority of microbial communities inhabiting surface sediments are proposed to be heterotrophs, utilizing organic matter to meet their carbon and energy demands. With continuous sedimentation over geologic time, the deposited organic matter is buried in the seafloor and becomes increasingly recalcitrant to microbial unitization [8, 15]. However, it has been proposed that specific community members can thrive on these recalcitrant compounds [17]. Sedimentary organic matter consists mainly of biological macromolecules, including carbohydrates, lipids, proteins, and nucleic acids, as well as other complex substances, such as humic and fulvic acids [10, 18, 19]. Generally, complex macromolecules are made bioavailable by extracellular enzymatic cleavage. The formed oligomers and monomers are fermented into smaller molecules that further feed sulfate reduction and methanogenesis. Biogenic methane gas can be an important source for gas hydrate formation and accumulation [1, 20, 21].

Subseafloor gas hydrates are vulnerable to dissociation under changing environmental conditions, e.g., rising ocean temperature or dropping hydrostatic pressure, leading to the emission of gases (mostly methane) into the ocean and the atmosphere [22, 23]. When in contact with sulfate, these gases supplied from deep reservoirs serve as alternative energy and carbon sources for benthic microbes. Within sulfate methane interfaces, methane is consumed by anaerobic methane-oxidizing archaea (ANME), typically forming syntrophic consortia with sulfate-reducing partner bacteria [24, 25]. In surface or shallow sediments, methane fluxes were reported to greatly stimulate the growth of ANME and coupled sulfate reducers [7, 26]. However, it remains unclear whether the degradation of organic matter or methane fluid is the main metabolic strategy to sustain microbial life in the deep subseafloor of the gas hydrate zone.

In this study, we explored microbial community compositions and their functions and activities for carbon and energy acquirements in the deep subseafloor of the gas hydrate zone. Four cores were drilled from the gas hydrate zone in the South China Sea. Through a combination of geochemical measurements, metagenomics, and metatranscriptomics, we show that fermentation has a dominant role in microbial metabolism, even in methane-rich habitats of the deep biosphere.

## Results

### Upward methane fluxes influence the geochemistry of sediments

Four cores from gas hydrate drilling sites (Figure S1; see the “Material and methods” section) were retrieved from the Shenhu area (*n* = 1; SH-W20A) and Qiongdongnan Basin (*n* = 3; QDN-W01B, W03B, and W04B). All four drilling sites were located above deep subsurface gas chimneys, indicating upward gas fluxes within sediments [27, 28]. All cores except QDN-W04B contained gas hydrates with various morphologies. The natural gases in the Shenhu area and Qiongdongnan Basin were reported to consist predominantly of methane accompanied by C_2_–C_5_ gases [29, 30]. Accordingly, methane and non-methane gaseous alkanes, including ethane and propane, were detected in these four cores (Table S1).

**Fig. 1:**
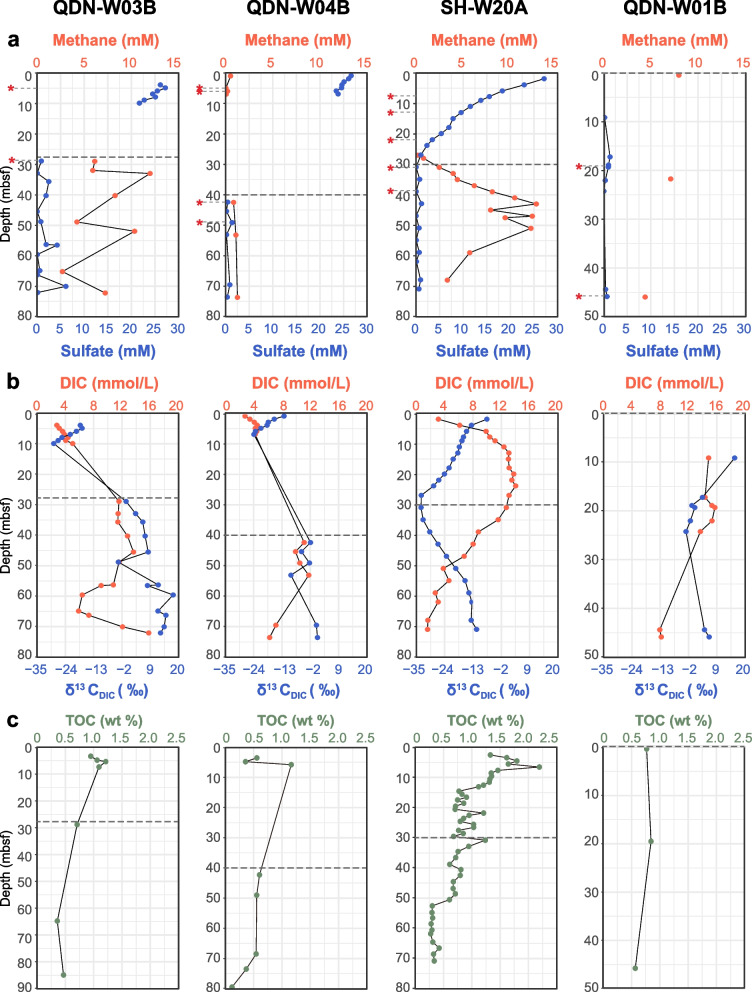
Sediment geochemistry of samples cored in the hydrate zone. Depth profiles of **a** methane and sulfate, **b** DIC contents and δ^13^C values of DIC, and **c** TOC contents. Dashed lines mark the predicted sulfate-methane interface predicted based on linear sulfate gradients. Samples used for metagenomic and metatranscriptomic analyses are marked with red asterisks

Based on porewater sulfate and methane profiles plus the extrapolation of linear sulfate gradients [31–33], we predicted their depth distributions of the sulfate-methane interface (SMI; Fig. 1a). For QDNB-W03B, QDNB-W04B, and SH-W20A, the SMI depths should be approximately 28, 40, and 30 mbsf, respectively [7, 33]. The DIC profiles also supported active sulfate reduction above the SMI, with increased DIC concentrations above the SMI and gradually decreasing DIC contents after sulfate depletion (Fig. 1b). In parallel, δ^13^C values of DIC were slightly negative at the top of the cores (− 7.6∼ − 18.2‰) and became more depleted with increasing sediment depth (− 23.9∼ − 33.2‰). This decrease may be caused by the oxidation of isotopically depleted methane. Below the depth of the SMI, δ^13^C_DIC_ values increased to − 11.6 ~  − 13‰, indicating the dominance of organic matter degradation (Fig. 1b). For these three cores, total organic carbon (TOC) contents in sediments decreased with depth (Fig. 1c), suggesting active microbial carbon mineralization [26, 34].

In contrast, QDN-W01B showed low sulfate concentrations (< 1.5 mM) in the entire sampled core (9 − 45 mbsf; Fig. 1a), and the concentrations of methane were in the range of a few millimoles. These porewater profiles indicated a shallow SMI near the sediment/water interface [7, 33]. At 9 − 45 mbsf of sediments in this core, TOC contents were observed to range between 0.57 and 0.85% (Fig. 1c). Simultaneously, DIC concentrations decreased, whereas δ^13^C_DIC_ values initially decreased and then increased with depth (Fig. 1b). These data suggested the potential occurrence of microbial fermentation at these depths considering the lack of availability of sulfate acceptors [34].

### Microbial communities are shaped by redox zonation

**Fig. 2:**
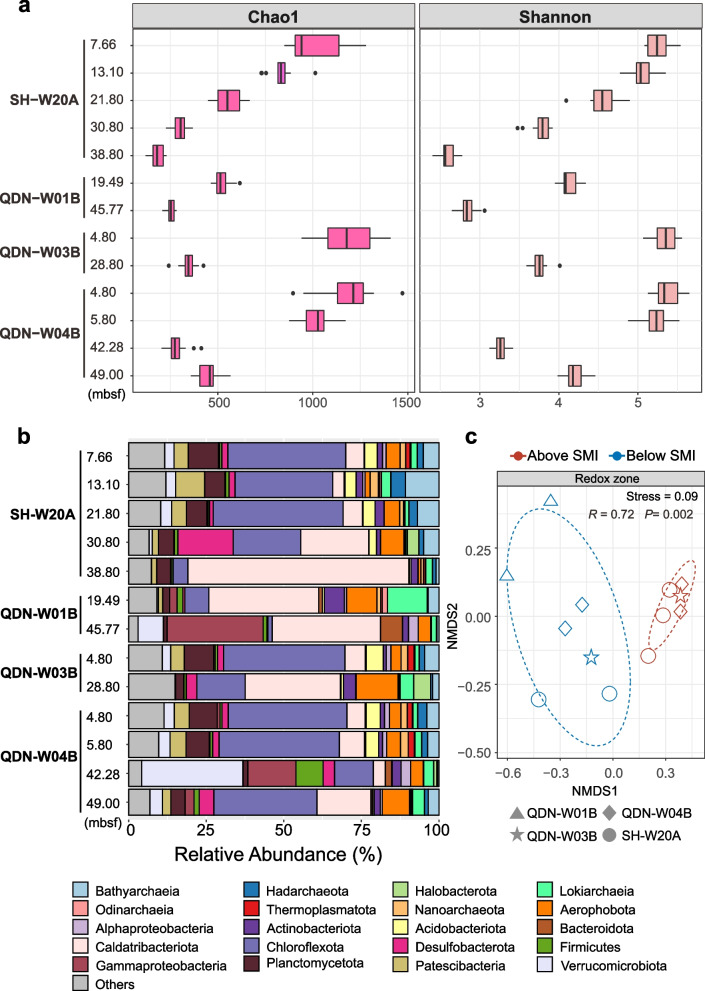
Microbial composition and diversity analysis of the deep subsurface sediments from the gas hydrate zone. **a** Alpha diversity of microbial communities based on metagenomic reads of 14 single-copy marker genes. **b** Taxonomic compositions of microbial communities based on 16S miTags extracted from the metagenomes. Full taxonomic information is provided in Table S2. **c** NMDS analysis of a Bray–Curtis dissimilarity matrix calculated from the single-copy marker gene *rplB* OTU table. The significance of the differences in the community structures of the different redox zones was tested using ANOSIM with 999 permutations

To compare deep subseafloor microbiomes in response to distinct redox zones, 13 sediment samples were selected for shotgun metagenomic sequencing based on porewater sulfate concentrations and available DNA yields. For each sediment core, the alpha diversity analysis based on Chao1 and Shannon indices determined on single-copy marker genes revealed a decline in microbial diversity with depth (Fig. 2a). When samples were grouped according to biogeochemical zonation, subseafloor sediments above the SMI supported significantly more diverse communities than those below the SMI (Wilcoxon rank sum test, *P* < 0.001; Figure S2). This is evidenced by higher Chao1 (966 ± 247 vs. 315 ± 113) and Shannon (5.1 ± 0.3 vs. 3.5 ± 0.6) values above the SMI (Fig. 2a), highlighting the importance of sulfate availability in shaping microbial community compositions.

To profile microbial communities, we classified 16S rRNA gene fragments (i.e., 16S miTags) recruited from shotgun metagenomic reads (Fig. 2b). In QDNB-W01B, Caldatribacteriota was the most abundant lineage (on average 35.2% of the whole community), followed by Gammaproteobacteria (16.7%), *Lokiarchaeia* (7.2%), and Aerophobota (6.9%). The microbial communities from QDNB-W01B were similar to those from sediments below the SMI in SH-W20A, QDNB-W03B, and QDNB-W04B (Fig. 2b). For sediments above the SMI in the latter group, Chloroflexota was the most abundant lineage (37.9%), followed by Planctomycetota (8.1%), Bathyarchaeia (5.8%), and Acidobacteriota (4.3%). The beta diversity analysis confirmed that deep subseafloor community variations were strongly correlated with redox zones (ANOSIM, *R* = 0.72, *P* = 0.002; Fig. 2c). Members of the Halobacterota phylum were observed to be abundant only in sediments below the SMI in SH-W20A (3.7%) and QDN-WO3B (5.5%), consisting of mostly ANME-1 and *Methanosarcina* (Figure S3).

### The most abundant microorganisms encode fermentation pathways

Metagenomic assembly and binning yielded 578 metagenome-assembled genomes (MAGs, < 99% ANI) with > 50% completeness and < 10% contamination (Table S3 and Figure S4). They clustered into 349 bacterial and archaeal species-level clades, with most having < 1% relative abundance across all samples (Figure S5 and Table S4). To estimate the genomic capabilities to grow on biological macromolecules, we screened these MAGs for the presence of genes encoding CAZymes, peptidases, and nucleases. In total, we detected 21,942 potential CAZymes in 577 MAGs (Table S5**)** and 46,693 potential peptidases in 578 MAGs (Table S6). Approximately 2.3% of CAZymes and 2.6% of peptidases could potentially be released into the environment (Tables S5 and S6). Secreted CAZymes and peptidases are important for the cleavage of polymeric substrates before their incorporation into cells [35].

**Fig. 3:**
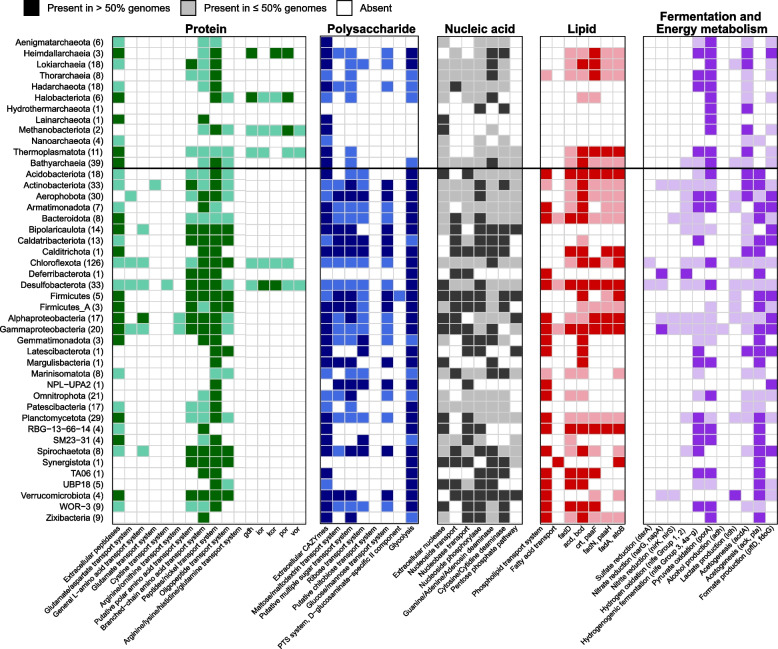
Presence of key genes in the degradation of biological macromolecules, fermentation, and energy metabolism detected across phylogenetic clusters. Dark and light color shading indicates gene presence in > 50% and 1–50% of the MAGs in each phylogenetic cluster, respectively. The number of MAGs per phylogenetic cluster is shown in brackets. The presence of a pathway was determined by the software METABOLIC. A complete list of the annotations is provided in Table S7

**Fig. 4:**
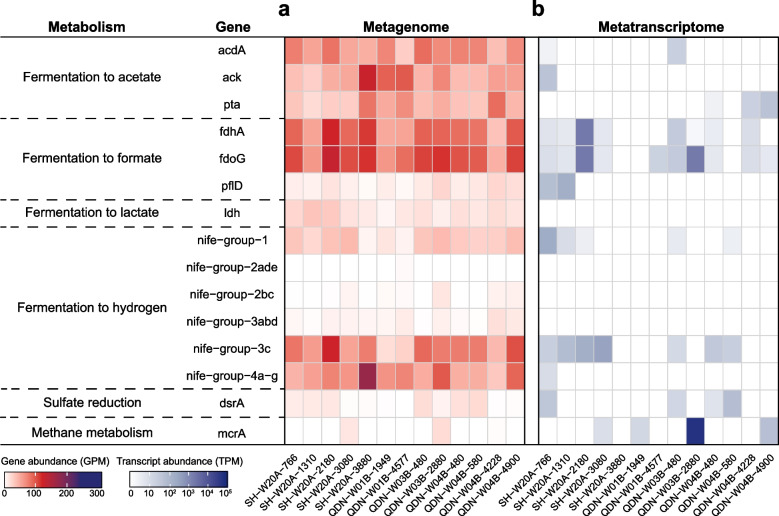
Abundance and expression of metabolic marker genes in the deep subsurface sediments from the gas hydrate zone. **a** The abundance of each gene in metagenomes. **b** The abundance of each transcript in the metatranscriptomes. Gene and transcript abundances are represented in units of genes per million (GPM) and transcripts per million (TPM), respectively

Of the 21,942 CAZyme hits (Figure S6), genes belonging to glycosyltransferases were the most abundant (46%), followed by glycoside hydrolases (32%), carbohydrate esterases (9%), and carbohydrate-binding modules (5%). The genes for carbohydrate degradation were concentrated in MAGs from phylogenetically diverse microbial phyla/classes, including the dominant bacterial and archaeal lineages of Chloroflexota, Caldatribacteriota, Verrucomicrobiota, Bathyarchaeia, *Lokiarchaeia*, and other phylogenetic clusters (Fig. 3 and Table S7). Most of these MAGs encoded diverse extracellular CAZymes that allow the cleavage of multiple carbohydrates and numerous sugar transport systems for oligo-/monomer uptake (Figure S7). Consequently, these microorganisms might be able to utilize a broad spectrum of carbohydrates, including chitin, cellulose, pectin, polyphenolics, starch, xylans, and xyloglucan (Table S8). The simple sugars produced by the activity of extracellular CAZymes can enter the glycolysis pathway, which is prevalent across bacterial and archaeal lineages (Fig. 3). Most bacterial and archaeal MAGs lack genes for respiration. Instead, they encoded the potential to metabolize pyruvate produced during glycolysis to acetyl-CoA and further into fermentation pathways (Fig. 3), yielding various organic acids. These acids mainly include acetate (*acdA* or *pta* + *ack*), formate (*fdoG* or *pflD*), and lactate (*ldh*). In addition, fermentative hydrogen production (Nife Group 3, Nife Group 4a-g) might function as another electron sink for the anaerobic degradation of macromolecules. Gene-centric surveys also showed genes encoding enzymes involved in formate (up to 163.18 genes per million, GPM), acetate (up to 158.81 GPM), lactate (up to 43.68 GPM), and hydrogen (up to 210.91 GPM) fermentation to be highly abundant (Fig. 4a).

A majority of the MAGs encoded extracellular peptidases and amino acid transporters (Fig. 3 and Figure S8). However, only a few lineages such as Chloroflexota, Desulfobacterota, and Heimdallarchaeia contained genes encoding enzymes for amino acid degradation (*gdh*, *por*, *vor*, *kor*, and *ior*; Fig. 3) and therefore could be able to use environmental proteins as energy sources. Most other MAGs lack such an amino acid degradation pathway.

The genomic potential to degrade DNA was distributed among MAGs affiliated with Chloroflexota, Desulfobacterota, Proteobacteria, and Bathyarchaeia (Fig. 3). These MAGs contained genes for extracellular nucleases, nucleoside/nucleobase transporters, and phosphohydrolases for cleaving nucleoside into the ribose and base moieties, along with pathways for purine and pyrimidine degradation. The released ribose could be further utilized via the pentose phosphate pathway (Fig. 3). The retrieved MAGs of Caldatribacteriota and Verrucomicrobiota did not encode genes for extracellular nucleases, but genes for nucleoside/nuclebase transporters and corresponding downstream pathways.

Genes coding enzymes for lipid degradation were less abundant in the sediments. Chloroflexota, Desulfobacterota, Proteobacteria, Bacteroidota, and Actinobacteriota were found to harbor catabolic potentials for lipid degradation (Fig. 3). They encoded phospholipid/fatty acid transporters and complete beta-oxidation pathways. The dominant lineage of Caldatribacteriota in the deeper subseafloor seems to lack most genes that encode mechanisms to import and metabolize lipids or fatty acids. Caldatribacteriota MAGs recovered from other anaerobic environments, e.g., petroleum reservoirs [36] and hot spring sediments [37], have also been shown to lack genetic potential for fatty acid degradation.

### Sulfate reducers and methanogens are rare

Only a few MAGs were identified as potential sulfate reducers. They belonged to Chloroflexota (*n* = 5) and Desulfobacterota (*n* = 10, Fig. 3). These sulfate reducers possess genes encoding a near complete dissimilatory sulfate reduction pathway (i.e., *dsrA*/*B*, *aprA*/*B*, and *sat*) and other key genes, including *dsrC* and *dsrMKJOP* (Figure S9). With the availability of sulfate in sediment samples above the SMI, the products of microbial fermentation, such as acetate, hydrogen, or C1-compounds, can be further oxidized by sulfate reducers to CO_2_. A quantitative assessment of *dsrA* genes suggested that potential sulfate reducers are solely detected in sediments above the SMI (up to 27.12 GPM, Fig. 4a).

The *mcrA* genes (Fig. 4a) were detected only in or below the SMI in QDN-W04B (up to 2.58 GPM), SH-W20A (up to 18.35 GPM), QDN-W03B (up to 25.79 GPM), and QDN-W01B (0.23 GPM). A total of seven recovered MAGs possessed genes encoding McrA. Taxonomic placements showed that these MAGs belonged to Heimdallarchaeia, Methanomassiliicoccales, and Methanofastidiosales, along with ANME-1 and ANME-2 (Figure S10). With the exception of Heimdallarchaeia SCS_cbin_173, each *mcrA*-containing archaeal MAG encoded a complete MCR complex (McrABG) located on a long contig (> 10 kbp) with nearby genes annotated as methane metabolism-related enzymes, as well as tRNAs and ribosomal proteins that were interspersed within the corresponding contig (Figure S11). Such an organization of genes was also previously reported in other Mcr-containing archaea [38]. Heimdallarchaeia SCS_cbin_173 only encoded a partial Mcr complex (McrAG) located on a short contig (3 kbp) without the nearby genes mentioned above (Figure S11). The low completeness (55.4%) of Heimdallarchaeia SCS_cbin_173 is likely responsible for the absence of these genes.

**Fig. 5:**
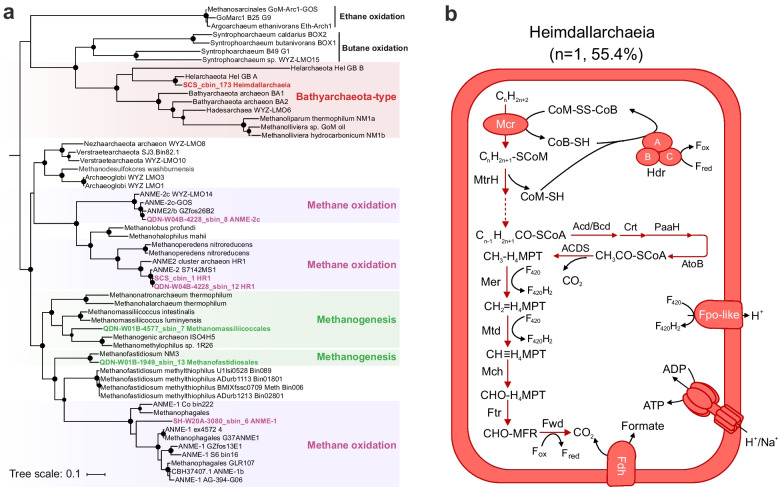
Methane and alkane metabolism. **a** Phylogenetic tree constructed based on alignments of McrA protein sequences. Black dots indicate bootstrap values of 70–100%. Scale bars indicate the average number of substitutions per site. **b** Anaerobic oxidation of multi-carbon alkanes. Dashed arrows indicate steps catalyzed by unconfirmed enzymes. The percentages between brackets indicate the estimated completeness of the corresponding MAGs. A complete list of metabolic information can be found in Table S11

Based on the McrA phylogeny, Mcr complexes in this study were categorized into two major groups (Fig. 5a), including (1) conventional Mcr clustering with known methanogens or ANME and (2) Mcr-like enzymes associated with archaea that are capable of the degradation of multi-carbon alkanes. Methanofastidiosales QDN-W01B-1949_sbin_13 and Methanomassiliicoccales QDN-W01B-4577_sbin_7 were closely related to cultured hydrogen-oxidizing methylotrophic methanogens [39, 40] based on both phylogenomic and McrA trees (Fig. 5a and Figure S10). These two near-complete MAGs (90.26–96.46%) lacked genes encoding the methyl-H_4_MPT:coenzyme M methyltransferase (Mtr) and the methyl-branch of the Wood–Ljungdahl pathway (Figure S12), which are present in all CO_2_-reducing methanogens [41]. Instead, they encoded methyltransferases (Figure S12) with the potential to support methanogenesis from methanol (MtaA) and methylamine (MtbA). These two hydrogen-dependent methylotrophic methanogen MAGs were found to be present at 19.49 and 45.77 mbsf in QDN-W01B with low abundances (0.09–0.11% of the communities; Figure S13 and Table S4).

### Anaerobic methanotrophs and alkanotrophs are also rare

Four MAGs affiliated with anaerobic methanotrophs ANME-1 and ANME-2 possessed conventional *mcr* genes, along with an archaeal Wood–Ljungdahl pathway for oxidizing methane to CO_2_ and HdrABC to recycle the CoM and CoB heterodisulfides (Fig. 5a and Figure S14). ANME-1 was found to be abundant in sulfate-depleted layers, i.e., at 30.8 mbsf in SH-W20A and 28.8 mbsf in QDN-W03B, with relative abundances ranging from 2.7 to 3.7% (Figure S13). ANME-2 was also found in sulfate-depleted layers, i.e., at 42.28 and 49.00 mbsf in core QDN-W04B, but with lower abundance than ANME-1 (0.2–1.3%; Figure S13). None of the ANME MAGs encoded any canonical terminal reductases (such as sulfate and nitrate reductases; Table S9), leading to the conclusion that syntrophic partners would be necessary to enable their growth on methane. However, no obvious syntrophic sulfate reducers were identified in these sulfate-depleted sediments (Figure S13). All ANME MAGs contained genes for multi-heme cytochromes (Table S10) that are often involved in direct electron transfer to a bacterial partner or iron/manganese oxides [42].

One Heimdallarchaeia MAG (SCS_cbin_173) contained *mcrA* sequences (Fig. 5a) that were clustered with homologs from recently discovered alkane-oxidizing Helarchaeales (namely, Helarchaeota in NCBI taxonomy) [43, 44]. The McrA from these two lineages formed a monophyletic clade (Fig. 5a) with those from the cultured multi-carbon alkane degraders *Candidatus* Syntrophoarchaeum [45], *Candidatius* Argoarchaeum ethanivorans [46], *Candidatius* Methanoliparium, and MAGs of microorganisms with inferred alkane metabolism, such as Bathyarchaeia [24, 47]. The Heimdallarchaeia MAG also encoded a HdrABC for the regeneration of the heterodisulfide CoM-S–S-CoB, methyltransferases to convert alkyl-CoM to acyl-CoA, a complete beta-oxidation pathway to oxidize acyl-CoA to acetyl-CoA, and an archaeal Wood-Ljungdahl pathway (Fig. 5b), a set of genes similar to genomic features found in Helarchaeales [43, 44]. The Heimdallarchaeia MAG present here contained genes encoding the Fpo complex for energy transfer across the cell membrane (Fig. 5b). The identified Heimdallarchaeia was rare among the four cores, comprising only 0.002–0.03% of the community (Figure S13). Like its Helarchaeales relatives, this Heimdallarchaeia MAG lacked internal electron sinks and multi-heme cytochromes, but contained formate dehydrogenases that could facilitate the transfer of reducing equivalents in the form of formate (Fig. 5b). Genes encoding hydrogenases were not identified in the genome of Heimdallarchaeia (Table S9), possibly related to its low genome completeness.

### Genes for macromolecule utilization and alkane oxidation are actively expressed

**Fig. 6:**
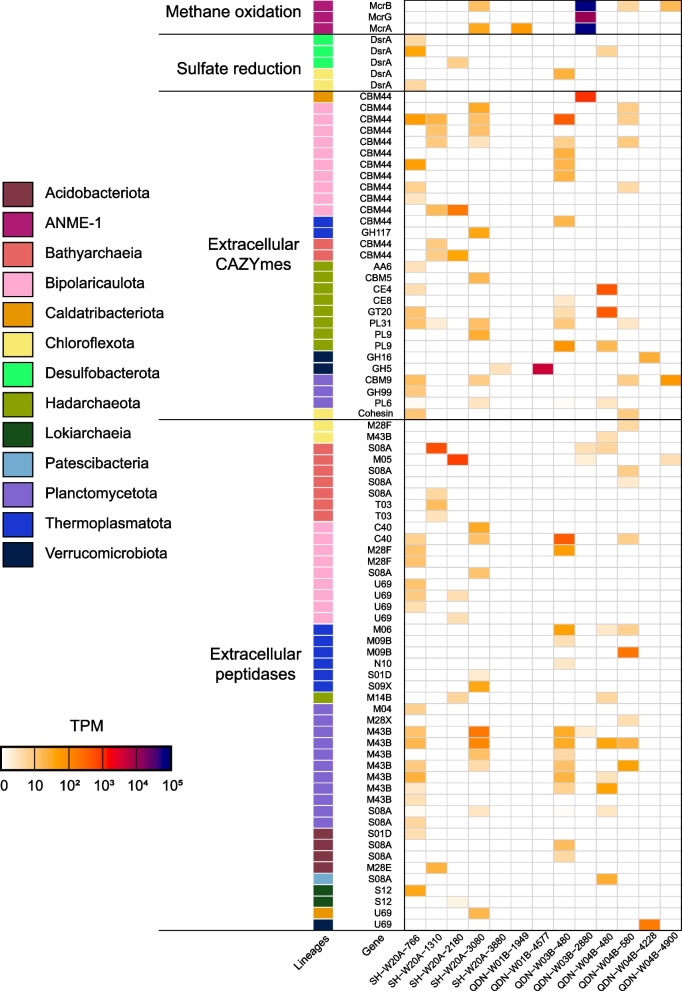
Transcription of genes for sulfate reduction, methane oxidation, and extracellular CAZymes and peptidases in the genomic bins. The expression levels of each gene are represented in units of transcripts per million (TPM). A complete list of the transcriptional information is represented in Table S12

Metatranscriptomic sequencing was performed on these samples to elucidate microbial activities for the recycling of macromolecules and alkanes. Genes encoding various extracellular CAZymes were transcribed across most samples. The transcripts encode the breakdown of pectin, chitin, amorphous cellulose, and polysaccharides (Fig. 6). These transcripts for extracellular CAZymes were expressed in Chloroflexota (up to 17 TPM), Caldatribacteriota (up to 2122 TPM), Verrucomicrobiota (up to 8411 TPM), Bipolaricaulota (up to 942 TPM), Planctomycetota (up to 147 TPM), Bathyarchaeia (up to 96 TPM), Thermoplasmatota (up to 88 TPM), and Hadarchaeota (up to 1113 TPM). Although very few transcripts of secretory CAZymes and peptidases were detected in QDN-W01B, we observed transcripts related to various CAZymes and peptidases located in the cell membranes or cell walls (cell-attached CAZymes and peptidases) in this core (Table S12). These cell-attached CAZymes and peptidases are also important for the hydroxylation of environmental macromolecules via tighter hydrolysis-uptake coupling, especially in the deep subseafloor environment where organic matter is refractory [35]. Transcripts that encode extracellular peptidases were found for Chloroflexota, Caldatribacteriota, Bipolaricaulota, Planctomycetota, Bathyarchaeia, Thermoplasmatota, *Lokiarchaeia*, and Hadarchaeota (Fig. 6). Both gene- and genome-resolved analyses revealed that genes involved in sulfate reduction (*dsrA*) were transcribed only in sediments above the SMI, with Desulfobacterota and Chloroflexota being major sulfate reducers (Figs. 4 and 6). This result also illustrated that Chloroflexota and Desulfobacterota in sediments above the SMI (Fig. 2) most likely actively degraded macromolecule-derived carbon coupled with sulfate reduction. When sulfate was depleted, transcripts assigned to formate, acetate, and hydrogen fermentation were found to be highly abundant, e.g., in SH-W20A and QDN-W03B (Fig. 4b).

Based on gene-level analysis, *mcrA* genes were expressed in and below the SMI zones (Fig. 4b), with the highest transcription in QDN-W03B at 28.8 mbsf (71,981 transcripts per million, TPM). Most of the *mcrA* transcripts mapped to the MAG ANME-1 SH-W20A-3080_sbin_6 (Fig. 6 and Table S12) in QDN-W03B (139,474 TPM at 28.8 mbsf), whereas this transcription was much lower in SH-W20A (81 TPM at 30.8 mbsf). Genes encoding beta (McrB) and gamma (McrG) subunits of the Mcr complex encoded in this genome were also expressed to different degrees. Notably, *mcrA* genes were hardly detected in the metagenomes and MAGs of QDN-W01B, and the transcriptome contained only a few *mcrA* reads.

## Discussion

This study combined geochemistry, metagenomics, and metatranscriptomics to characterize deep subseafloor microbial community structures and carbon metabolism in sediments experiencing methane fluxes. Methane fluxes strongly reflect the geochemistry of porewaters along depth in the Shenhu area and Qiongdongnan Basin. A shallow depth of SMI was observed in QDN-W01B corresponding to rapid sulfate depletion, which may be attributed to high methane fluxes [7]. In SH-W20A, QDN-W03B, and QDN-W04B, low methane fluxes allowed sulfate to diffuse into greater depths.

The alpha analysis showed that microbial diversities in deep subseafloor sediments decreased with increasing sediment depth. The phenomenon of depth-related declines in microbial diversity is also reported by a previous study that investigated global marine sediments [48]. Metagenomic profiling revealed that microbial community compositions in deep subseafloor sediments were significantly correlated with redox zonation. In subseafloor layers above the SMI, members of Chloroflexota predominated, while members of Caldatribacteriota were dominant in subsurface sediments below the SMI. Chloroflexota and Caldatribacteriota are broadly defined taxonomic groups and have been found to co-occur at high abundances in sediments from many different regions across global oceans [48]. The transition from Chloroflexota to Caldatribacteriota between redox zones along with sediment depth implies intense subseafloor selection [49]. Members of Caldatribacteriota are likely more adaptive to deep anoxic sediments with less organic carbon and energy availability [3, 50], which explains their successful survival in the deep subseafloor.

Genome-based functional analysis suggests that microbial communities utilize biologically produced macromolecules (mainly carbohydrates) as carbon and energy sources regardless of the availability of methane. The main heterotrophs are Chloroflexota, Caldatribacteriota, Verrucomicrobiota, Hadarchaeota, and Bathyarchaeia. These lineages were also observed to conduct heterotrophic fermentative metabolisms in common marine sediments not influenced by methane fluxes. For example, metatranscriptomics and enzyme assays suggested that Caldatribacteriota actively catabolized sugars and proteins in the subsurface sediments of the Baltic Sea [51]. Verrucomicrobiota and Chloroflexota were also found to be dominant heterotrophs in sediments of the Helgoland mud area [15]. Most of these microorganisms seem to be generalists that degrade a wide range of biopolymers. The majority of the deep subseafloor communities could extracellularly hydrolyze macromolecules by secreting enzymes into the surrounding environment and take up oligomers via various transporters. Genes encoding CBM44, which is involved in cellulose and xyloglucan binding [52], were widely transcribed in the deep subseafloor communities. Cellulose and xyloglucan are derived from terrestrial vascular plants [53]. In short, our results demonstrate the degradation of plant matter in deep subseafloor sediments of the gas hydrate zone.

Nucleic acids represent another key trophic resource and provide an energy source [54], but their role in sedimentary biogeochemical cycles has largely been ignored to date. The catabolism of nucleic acids is involved in extracellular cleavage and transmembrane import before intracellular degradation [18, 19]. Many Caldatribacteriota and Verrucomicrobiota contain nucleoside/nucleobase transporters for the uptake of these compounds, but they lack extracellular nucleases such as the Nuc superfamily (DNA/RNA nonspecific endonucleases) [55]. These microorganisms act as opportunists that depend on other heterotrophs that produce exoenzymes. Such co-occurrence of exoenzyme-producing and opportunistic scavengers seems to be common in nature and has, for instance, been observed in Guaymas Basin hydrothermal sediments [18] and terrestrial soils [56].

The products of fermentation (formate, lactate, acetate, and hydrogen) can be further oxidized into CO_2_ by other microbes using sulfate as an electron acceptor in the sediment layers above the SMI or used for methanogenesis when sulfate is depleted. In addition to the typical sulfate reducers of Desulfobacterota [57], the presence of *dsrAB* genes in several MAGs (*n* = 5) of Chloroflexota suggested their ability to perform sulfate respiration. Bacteria of Chloroflexota are widespread and highly abundant in various marine sediments [50] and display a broad spectrum of metabolic traits, e.g., reductive dehalogenation and fermentation of organic matter [58, 59]. Only in recent years have their roles in the deep subsurface sulfur cycle been recognized [60]. Our data expand the limited number of Chloroflexota genomes, which may provide important information for the cultivation of deep-sea sulfate-reducing Chloroflexota. Although the conditions in the subseafloor were considered to be suitable for hydrogenotrophic or acetoclastic methanogenesis, we only detected H_2_-dependent methylotrophic methanogens (Methanomassiliicoccales and Methanofastidiosales), which produce methane using hydrogen and C1 substrates and synthesize biomass using acetate [61]. Similarly, in other reports about sediments of the JiaoLong, Shenhu, and Haima methane seeps from the South China Sea as well as hydrate-bearing sediments from the eastern Nankai Trough, H_2_-dependent methylotrophic methanogenesis was found to be a major methanogenic pathway [62–65]. Methylotrophic methanogens utilize non-competitive substrates (methanol, methylamines, methyl sulfide, and related compounds) originating from the degradation of organic macromolecules such as lignin and pectin [66]. Therefore, the fermentation of macromolecules does not necessarily support hydrogenotrophic or acetoclastic methanogens but can also be linked to H_2_-dependent methylotrophic methanogenesis.

The influx of methane from the deep serves as an energy source for ANME archaea, but these make up only a small percentage of the microbial communities. Combined metagenomic and metatranscriptomic evidence suggested that higher gene and transcript abundances for *mcrA* genes were found in sediments below the SMI of SH-W20A, QDN-W03B, and QDN-W04B. ANME-1 (up to 3.7% of the whole community) and ANME-2 (up to 1.3%) were found to be the main methane oxidizers in sediments below the SMI, with ANME-1 being highly active based on expressed transcripts. The observed response of ANME communities in the deep subseafloor is different from that of ANME communities fueled by high methane fluxes in the sulfate-rich surface sediments from Arctic seafloor gas hydrate mounds [7]. Previous observations of ANME-1 and ANME-2 clades have focused mainly on shallow sediments at no more than 20 mbsf, in the sulfate-methane transition zones (SMTZ) or occasionally sulfate-depleted horizons below the SMTZ [7, 63, 67–69]. The results presented here indicate that ANME archaea are still active in deeply buried marine sediments with depths over 30 mbsf. Despite in sulfate-depleted subseafloor layers, the presence of genes for multi-haem cytochromes in these ANME archaea suggests that they might transfer electrons to solid metal oxides (e.g., Fe and Mn) other than sulfate for energy conservation [70], coupled with oxidizing methane that migrates from deeper reservoirs through gas chimneys.

One Heimdallarchaeia MAG stood out particularly because it contained alkyl-CoM reductase and downstream parts for the utilization of short-chain alkanes. Despite belonging to the rare biosphere (up to 0.03% of the community), Heimdallarchaeia may play a substantial role in the community in response to environmental oxidation of non-methane alkanes. Mcr-based methane cycling was thought to be limited to Halobacteriota (namely Euryarchaeota) [71]. Until recently, non-Halobacteriota phyla, including the TACK superphylum and Helarchaeales, have been shown to contain proteins with homology to Mcr [43, 47, 72]. Helarchaeales is so far the only known group of Asgard archaea genetically capable of Mcr-mediated hydrocarbon oxidation. Based on the phylogenetic analysis, *mcrA* genes from Heimdallarchaeia and Helarchaeales are closely related. Furthermore, both Heimdallarchaeia and Helarchaeales lack genes for canonical pathways of sulfate reduction and cytochromes. They hence most likely need external partners to consume the reducing equivalents in the forms of hydrogen and formate [43, 44]. The *mcr* genes in Helarchaeales were previously thought to be acquired from, e.g., Bathyarchaeia or *Syntrophoarchaeum* due to horizontal gene transfer events. Within the Asgard archaea, these genes are restricted to Helarchaeales [43]. The discovery of Heimdallarchaeia with potential for multi-carbon alkane degradation indicates a broader view of the evolution and expansion of hydrocarbon oxidation pathways within Asgard archaea [43, 44, 73, 74].

## Conclusion

Together, our results uncover the metagenomic blueprints and metatranscriptomic activities of microbes residing in deep subseafloor sediments from the gas hydrate zone. The sulfate above the SMI sustains small numbers of sulfate reducers. The upward-migrating methane does not always provide energy and carbon sources for microbial communities due to the lack of suitable electron donors in gas hydrate sediments. More importantly, these findings underpin that fermentation of macromolecules sustains a large number of different archaea and bacteria in deep subseafloor sediments. Microorganisms that use fermentation products for methanogenesis or sulfate reduction are of minor abundance, and their marker genes, such as *mcr* or *dsr*, are expressed at low levels. This shows that energy conservation is concentrated in fermentative microorganisms. Although the fermentation reaction cannot be directly recognized by porewater profiles, in the studied sediments, the fermentative microorganisms outnumber the sulfate reducers, methane oxidizers and methanogens.

## Material and methods

### Sampling sites

The studied sediments were drilled from the Qiongdongnan Basin and Shenhu area (Figure S1), where considerable amounts of gas hydrates were discovered [21, 75, 76]. The Qiongdongnan Basin is an oil-bearing, fault-depression structural basin on the northwestern continental shelf of the South China Sea [21]. The Shenhu area is in the middle of the northern slope of the South China Sea and is tectonically located in the Pearl River Mouth Basin [75]. Four cores penetrating to 100 − 188 mbsf were analyzed in this study, with water depths ranging from 1000 to 1500 m. They were obtained during the 2019 gas hydrate drilling expedition (GMGS6) conducted by the Guangzhou Marine Geological Survey. Detailed descriptions of the seismic data, cores, and grain sizes of these samples were published previously [27]. The three drilling sites of cores QDN-W01B, QDN-W03B, and QDN-W04B were located in the Qiongdongnan Basin, and the core SH-W20A was recovered from the Shenhu area. Each sediment core was sectioned using a core extruder onboard immediately following retrieval. Samples were then frozen at − 80 °C for subsequent geochemical analysis and nucleic acid extraction.

### Geochemical measurements

Porewater geochemistry and total organic matter content were analyzed for 67 samples at depths of approximately 1 to 79 mbsf, and 45 samples ranging from 0.5 to 73.6 mbsf were selected for headspace gas analysis, following methods reported in our previous studies [73, 77]. Briefly, methane concentrations were measured using a headspace equilibration technique by an Inficon Fusion MicroGC gas chromatograph with a molecular sieve, PLOT Q columns, and thermal conductivity detectors. Sulfate concentrations were determined using ion chromatography (Metrohm 790 Personal IC). The concentrations and δ^13^C values of porewater DIC were analyzed via continuous flow mode-isotope ratio mass spectrometry (CF-IRMS). The TOC contents of bulk sediment samples were quantified on an elemental analyzer-isotope ratio mass spectrometer (EA-IRMS) after the removal of inorganic carbon.

### DNA extraction and metagenomic sequencing

Genomic DNA was extracted from approximately 10 g of sediments for each depth using the PowerMax Soil DNA Isolation Kit (Qiagen) according to the manufacturer’s instructions. DNA concentrations were evaluated using a Qubit 4.0 Fluorometer (Thermo Fisher Scientific). Metagenomic libraries were prepared for 13 samples following the manufacturer’s instructions (Illumina Inc.). Sequencing was performed on an Illumina NovaSeq 6000 platform with a 2 × 150 bp paired-end run at Berry Genomics Co. Ltd., Beijing.

### Metagenomic assembly and binning

Raw reads derived from the 13 metagenome libraries were quality-controlled using the Read_qc module (-skip-bmtagger) within the metaWRAP v1.2.2 pipeline [78]. Quality-controlled reads were assembled individually using metaSPAdes v3.13.0 [79] and co-assembled using Megahit v1.1.3 [80] within the metaWRAP Assembly module, producing 14 assemblies. Each assembly was binned using the Binning module (parameters: -maxbin2 -concoct -metabat2) and consolidated using the Bin_refinement module (parameters: -c 50 -x 10) within the metaWRAP pipeline. All binning results were aggregated and de-replicated using dRep v2.5.4 [81] at 95% and 99% average nucleotide identities, for species and strain levels, respectively. Completeness, contamination, and heterogeneity of MAGs were estimated using CheckM v1.0.18 [82].

### Taxonomic assignments of MAGs

The taxonomy of each MAG was assigned using GTDB-Tk v1.3.0 with reference to GTDB 05-RS95 [83]. The assignments were confirmed by the visual inspection of taxonomic trees. Reference genomes accessed from NCBI GenBank and the MAGs from this study were used to construct the phylogenomic tree based on concatenation of 43 conserved single-copy genes extracted by CheckM v1.0.18 [82], following the procedures reported in our previous study [14]. The maximum-likelihood phylogenomic tree was built using RAxML v8 with the PROTCATLG model, bootstrapped with 1000 replicates [84].

### Diversity and community profiling

Alpha and beta diversity of microbial communities were carried out using vegan package v2.5. Operational taxonomic units (OTUs) were extracted from the metagenomic data using SingleM v0.12.1 (https://github.com/wwood/singlem) by aligning to a database of 14 single-copy ribosomal proteins [85]. OTU tables were then summarized by rarefying and clustering using SingleM summarise. The Shannon index and Chao1 were calculated from the SingleM OTU tables across each of the 14 single-copy marker genes using vegan package v2.5. For beta diversity analysis, Bray–Curtis dissimilarity generated from the *rplB* OTU table was visualized using non-metric multidimensional scaling (NMDS) plots. A pairwise analysis of similarities (ANOSIM) was used to test for significant differences in community similarity between redox zones.

Microbial community structures were determined using both gene- and genome-centric approaches. For better community profiling in gene-centric analyses, 16S rRNA gene fragments (i.e., 16S miTags) were extracted from metagenomic raw reads using the phyloFlash v3.4 pipeline (parameters: -almosteverything) and classified with the SILVA v138.1 database [86]. For genome-centric analyses, the relative abundance of MAGs depreciated at species level was calculated using CoverM v0.4.0 (https://github.com/wwood/CoverM) with the genome mode (parameters: -min-read-percent-identity 0.95 -min-read-aligned-percent 0.75 -trim-min 0.10 -trim-max 0.90).

### Functional annotations

For all contigs assembled from 13 metagenomic samples, functional annotation was undertaken with METABOLIC v4.0 [87]. All predicted coding sequences were pooled and clustered at 95% nucleotide sequence similarity using CD-HIT v4.8.1 [88] (parameters: -c 0.95 -T 0 -M 0 -G 0 -aS 0.9 -g 1 -r 1 -d 0). Finally, a total of 3,701,930 non-redundant gene clusters were obtained and used as the reference gene catalog for microbial communities. The program Salmon v1.5.0 [89] in the mapping-based mode (parameters: -validateMappings -meta) was used to calculate gene abundance from the reference gene catalog in different metagenomes. Gene abundances were expressed as genes per million (GPM).

For individual MAGs, metabolic genes were identified by METABOLIC v4.0 [87]. Genomes were also annotated using DRAM with default parameters [90] against the KOfam, MEROPS, and dbCAN databases to identify CAZymes, peptidases, lipases, nucleases, transporters, and other proteins of interest. For cytochrome C detection, MAGs with the identified *mcrA* gene were screened for proteins based on characteristic cytochrome C CXXCH domains following the criteria described elsewhere [67]. Protein localization for CAZymes, peptidases, and cytochrome C was determined using the web tool Psortb v3.0.3 [91].

### Phylogenies of functional genes

For each gene, amino acid sequences from the current study were aligned with reference sequences using MAFFT v7.471 [92] (–auto option) and trimmed using TrimAl v1.2.59 [93] (–gappyout option). Maximum likelihood trees were constructed using IQ-TREE v2.0.5 [94], implemented in the CIPRES web server, with best-fit models and 1000 ultrafast bootstrap.

### Metatranscriptomic analysis

Total RNA was extracted from replicate samples of the metagenome analysis using the RNeasy PowerSoil Total RNA kit (Qiagen) according to the manufacturer’s instructions. RNA purity and concentration were evaluated using Qubit (Thermo Fisher Scientific). RNA integrity was accurately determined using the Agilent 4200 system (Agilent Technologies). Whole transcriptome amplification of total RNA was carried out using the RNA REPLI-g Cell WGA & WTA Kit (Qiagen) according to the manufacturer’s protocol. To enrich messenger RNA (mRNA), ribosomal RNA was depleted from total RNA using the ALFA-SEQ rRNA Depletion Kit. Whole mRNAseq libraries were generated by Guangdong Magigene Biotechnology Co. Ltd. (Guangzhou, China) using the NEBNext Ultra Nondirectional RNA Library Prep Kit for Illumina (New England Biolabs) following the manufacturer’s recommendations. The constructed libraries were sequenced on an Illumina NovaSeq 6000 platform and 150 bp paired-end reads were generated.

Raw metatranscriptomic reads were quality filtered in the same manner as metagenomes. The reads corresponding to ribosomal RNAs were removed using SortMeRNA v.4.2.0 [95]. Subsequently, these high-quality metatranscriptomic reads were mapped to the predicted protein-coding genes from all the MAGs and the reference gene catalog using Salmon v.1.5.0 [89] in mapping-based mode (parameters: -validateMappings -meta). The expression level for each gene was normalized to transcript per million (TPM).

## Supplementary Information

**Additional file 1: Figure S1.** Location of sampling sites in the gas hydrate zone of Shenhu area (SH) and Qiongdongnan (QDN) Basin from the South China Sea.All the four drilling sites werelocated above deep subsurface gas chimneys. They wereretrieved from the Shenhu area (*n =*1; SH-W20A) and Qiongdongnan Basin (*n =*3; QDN-W01B, W03B and W04B). The drilling depths of sediments are between 100and 188 mbsf in water depths ranging from 1000 to 1500 meters. **Figure S2. **Comparison of Chao1 and Shannon indices of the microbial community between sedimentsabove and below the SMI in the gas hydrate zone. Chao1 and Shannon indices werecalculated based on the SingleM ((https://github.com/wwood/singlem) OTU tablesfor 14 universal single-copy genes. P-values of differences between redox zones were calculated using Wilcoxon rank sum test. Asterisks denote significance (*** for *P* < 0.001). **Figure S3.** Relative abundances of each class from the Halobacterota phylum inferredfrom 16S rRNA gene fragments in metagenomes. **Figure S4.** MAG recovery information across differenttaxonomic levels. A Sankey diagram based on assigned GTDB taxonomy showing archaeal (a) and bacterial (b) MAGs at different phylogenetic levels. Numbers indicate the number of MAGs recovered for this lineage. (c) Total MAGs unclassified by GTDB-Tk at each taxonomic level. MAGs were dereplicated at strain level (i.e., 95% ANI). Detailed statistics for 578 MAGs are provided in Table S3. **Figure S5.** Phylogenetic placement of 349 MAGs for microbial communities in the deep subsurface sediments from the gas hydrate zone. Branches in red indicate unclassified MAGs. The maximum-likelihood phylogenomic tree was built based on concatenated amino acid sequences of 43 conserved single-copy genes using RAxML with the PROTCATLG model. The scale bar represents the average number of substitutions per site. Relative abundances of those microorganisms can be found in Table S4. **Figure S6. **Carbohydrate-active enzyme composition of the deep subseafloor microbiome from the gas hydrate zone. GTs: glycosyltransferases; GHs: glycoside hydrolases; CEs: carbohydrate esterases; CMBs: carbohydrate-binding modules; AA: auxiliary activities; PLs: polysaccharide lyases. **Figure S7.** Percentage of extracellular carbohydrate-activeenzymes (CAZymes) encoded in each phylogenetic cluster. The number of MAGs per phylogenetic cluster is shown in brackets. CMBs: carbohydrate-binding modules; CEs: carbohydrate esterases; GHs: glycoside hydrolases; GTs: glycosyltransferases; PLs: polysaccharide lyases. **Figure S8.** Percentage of extracellular peptidases encoded in each phylogenetic cluster. The number of MAGs per phylogenetic cluster is shown in brackets. **Figure S9.** Presence and absence of genes in sulfate-reducing pathway for recovered sulfate reducers reported in this study. **Figure S10.** Phylogenomic placement of MCR-containing MAGs based on 43 conserved protein sequences. Black dots indicate bootstrap values of 70–100%. Scale bars indicate the average number of substitutions per site. **Figure S11.** Co-localization of *mcr* with genes nearby. Genes are colored according to their functions. Red: Mcr complex; Purple: Methane/Alkane metabolism-related; Brown: Methanol utilization; Pink: Methylamine utilization; Orange: ABC transporter; Green: Cas protein; Greenyellow: tRNA/rRNA; Grey: Others. **Figure S12.** Predicted pathways of hydrogen-dependent methylotrophic methanogenesis. Grey colors indicate the absence of the enzyme or pathway. mWL pathway, methyl-branch of the Wood–Ljungdahl pathway. The percentages between brackets indicate the estimated completeness of the corresponding MAGs. A complete list of metabolic information can be found in Table S11. **Figure S13. **Relative abundances of hydrocarbon-metabolizing archaea and sulfate reducers inferred from metabolic pathway reconstructions. The MAG with the highest genome quality from each species cluster is picked as the representative. Details on species clusters and their relative abundances are presented in Table S4. **Figure S14.** Predicted pathways of anaerobic oxidation of methane. Grey colors indicate the absence of the enzyme or pathway. The percentages between brackets indicate the estimated completeness of the corresponding MAGs. A complete list of metabolic information can be found in Table S11.

**Additional file 2: Table S1.** Hydrocarbon concentrations at the Qiongdongnan basin and Shenhu area from the gas hydrate zone of the South China Sea. b.d. indicates below detection (0.02 mM). **Table S2.** Microbial compositions based on 16S miTags inferred from metagenomes. **Table S3.** Genome statistics of recovered archaeal and bacterial genomes. **Table S4.** Relative abundances of microorganisms based on representative MAGs for each species cluster. The relative abundances (%) were determined by mapping each MAG against quality-filtered metagenome reads using CoverM. **Table S5.** Total number of carbohydrate-active enzymes (CAZYmes) detected in 578 genomes. CAZYmes were identified by searching against the dbCAN datebase, using Distilled and Refined Annotation of Metabolism (DRAM). Subcellular localization was determined using PSORTb. **Table S6.** Total number of peptidases detected in 578 genomes. Peptidases were identified by searching against the MEROPS datebase. Subcellular localization for individual peptidase was determined using PSORTb. **Table S7.** Details for identification of genes involved in macromolecules degradation based on presence/absence matrix. **Table S10.** Summary annotations of carbohydrate degradation for each genomes using Distilled and Refined Annotation of Meabolism (DRAM). **Table S9.** Functional annotation for each genome using METABOLIC. **Table S10.** Details for identification of multiheme c-type cytochromes in McrA-containing MAGs. Subcellular localization was determined using PSORTb. **Table S11.** Details for identification of genes in genomes containing *mcrA* genes based on presence/absence matrix. **Table S12.** Expression of genes involved in methane/macromolecules degradation and sulfate reduction in 578 MAGs, in the unit of Transcripts Per Million (TPM).

## Data Availability

All metagenomic and metatranscriptomic raw reads used in this study are available under accessions SAMN19768611-19768623 (BioProject PRJNA739005). The assemblies, reference gene catalog, all MAGs, and phylogenetic trees can be found in figshare (https://figshare.com/s/0d2502c44a97bdd5133f).
